# Suppression of Induced microRNA-15b Prevents Rapid Loss of Cardiac Function in a Dicer Depleted Model of Cardiac Dysfunction

**DOI:** 10.1371/journal.pone.0066789

**Published:** 2013-06-19

**Authors:** Sashwati Roy, Jaideep Banerjee, Surya C. Gnyawali, Savita Khanna, Guanglong He, Douglas Pfeiffer, Jay L. Zweier, Chandan K. Sen

**Affiliations:** Davis Heart and Lung Research Institute and Department of Surgery, The Ohio State University Medical Center, Columbus, Ohio, United States of America; National Institutes of Health, United States of America

## Abstract

**Background:**

Dicer endonuclease, critical for maturation of miRNAs, is depleted in certain forms of cardiomyopathy which results in differential expression of certain microRNAs. We sought to elucidate the mechanisms underlying the rapid loss of cardiac function following cardiac-specific Dicer depletion in adult mice.

**Results:**

Conditional Dicer deletion in the adult murine myocardium demonstrated compromised heart function, mitochondrial dysfunction and oxidant stress. Elevated miR-15b was observed as an early response to Dicer depletion and was found to silence Pim-1 kinase, a protein responsible for maintaining mitochondrial integrity and function. Anti-miRNA based suppression of induced miRNA-15b rescued the function of Dicer-depleted adult heart and attenuated hypertrophy.

**Conclusions:**

Anti-miRNA based suppression of inducible miRNA-15b can prevent rapid loss of cardiac function in a Dicer-depleted adult heart and can be a key approach worthy of therapeutic consideration.

## Introduction

Arrest of miRNA maturation by targeted deletion of *Dicer* is known to perturb cardiac development [1], [2] and cause pathological consequences [3], [4]. Significance of such observations is heightened by the report that Dicer deficiency is associated with specific forms of cardiomyopathy and heart failure in humans [4]. We sought to develop an understanding of the pivotal pathways that initiate cardiac failure in response to targeted *Dicer* deletion in the adult murine heart. Our strategy has been to delineate primary causative mechanisms by focusing on early onset changes in the adult heart following *Dicer* deletion, with the goal to be able to rescue using miRNA-directed intervention. This approach resulted in the identification of elevated miRNA-15b in the heart as a causative factor. MiRNA-15b was found to silence Pim-1 kinase, required for mitochondrial integrity and cardio protection. Taken together with the observation that most types of human heart failure are associated with elevated miRNA-15b [5], [6], our findings recognize mitochondrial protection as a key mechanism in miR-15b targeted therapeutic approach for cardio protection.

## Materials and Methods

### Materials

The following materials were obtained from the sources indicated. Mice homozygous for *Dicer*-floxed (loxP) alleles were provided by Dr. Elaine Fuchs, The Rockefeller University, NY; B6129-Tg (Myh6-cre/Esr1)1Jmk/J (Jackson Laboratories); ethanol, corn oil, tamoxifen, 2, 2, 5, 5-tetramethyl-3-carboxylpyrrolidine-*N*oxyl (PCA); fetal bovine serum, complete Claycomb medium, fibronectin, gelatin, norepinephrine; L-ascorbic acid (Sigma Chemical Co., Milwaukee, WI); HL-1 cells were provided by William C. Claycomb., Louisiana State University, New Orleans, LA; antibiotic (penicillin, streptomycin), tetramethylrhodamine methyl ester (TMRM) (Invitrogen Corporation, Carlsbad, CA); culture dishes (Nunc, Denmark); mirVana™ miRNA isolation kit, SYBR green-I (Ambion, Austin, TX); Taqman assays and Taqman Universal master mix (Applied Biosystems, Foster City, CA); FlexmiR™ MicroRNA Labeling Kit (Luminex,Austin,TX); JC-1 dye (Molecular Probes, Eugene, OR); anti-Pim1 antibody, anti-Dicer, anti-ANT1(Abcam, Cambridge, MA), anti-ANF (LS Biosciences,Seattle, WA); DharmaFECT 1 transfection reagent, mmu-miRNA-15b mimic and mimic control (Dharmacon RNA Technologies, Lafayette, CO); lactate assay kit-K627-100 (Biovision Research Products, CA); secondary anti-Rabbit IgG (Amersham Pharmacia Biotech, Piscataway, NJ); MDA standards (TBARS assay kit, ZeptoMetrix Corp., NY); Anti-mmu-miR-15b Custom LNA™ Oligonucleotide (Exiqon Inc., MA); Pim1–3′UTR plasmid (Signosis, Sunnyvale, CA); dual-luciferase reporter assay system (Promega, Madison, WI) miRCURY LNA™ microRNA Inhibitor Negative Control A (Exiqon Inc., MA).

### Methods

#### Development of the transgenic mice

Mice homozygous for *Dicer*-floxed (loxP) alleles and transgenic B6129-Tg (Myh6-cre/Esr1)1Jmk/J were crossed to generate double transgenic (Myh6-cre/Esr1-Dicer^fl/fl^) mice. Adult male mice at 8 weeks of age (Myh6-cre/Esr1-Dicer^fl/fl^ and control Dicer^fl/fl^) were treated with vehicle (10%/90% vol/vol ethanol/corn oil) or tamoxifen (20 mg/kg body weight per day diluted in 10%/90% vol/vol ethanol/corn oil) by daily intraperitoneal injections for consecutive 5 days. Mice were housed in the institutional animal facilities with a 12-h light/dark cycle and controlled temperature, and allowed access to food and water *ad libitum*.

#### Measurement of cardiac function

MRI and echocardiography image acquisition and analysis was performed on all mice at the age of 8 weeks (17–21 g). MRI and echocardiography image acquisition and analysis was performed on all mice at the age of 8 weeks (17–21 g) as described previously [7]. *MR Imaging* : Briefly, mice were anesthetized in an anesthetic chamber using 1.5% isoflurane in 95% oxygen and 5% carbon dioxide mixture. MRI set-up experiments were carried out on a Bruker 11.7T (500 MHz) MR micro-imaging system comprising a vertical magnet of bore size 30 mm (Bruker Biospin, Ettlingen, Germany). Quadrature-driven birdcage coils with inner diameters of 25 mm (Bruker Biospin, Ettlingen, Germany) were used to transmit/receive the NMR signals. Mice were imaged using a 30-mm1H-imaging probe after scouting for long- and short-axis orientation of the heart, using a k-space segmented cardiac-triggered and ECG gated FLASH sequence. Five-six slices (slice thickness 1 mm) were then acquired in short-axis orientation covering the entire heart. The imaging parameters: FOV (30 mm^2^), matrix size 256×192 (256×256 reconstructed post imaging), TE/TR  = 1.43/8 ms, NEX  = 4, a flip angle of 15° and a Gaussian excitation pulse were applied in all experiments. *Echocardiography experiments* : Mice were lightly anaesthetized using 1.5% isoflurane in 95% oxygen and 5% carbon dioxide mixture, resulting in an average heart rate of 400 beats/min. A linear transducer was used for all the examinations as previously described [8]. The maximum penetration depth of this transducer is 1.2 cm with a centre focus at 6 mm. Ultrasound transmission gel was applied liberally on the anterior chest wall before scanning. A right parasternal short-axis view was used to visualize the cross-sectional view of left ventricle (LV). By inspecting the cine loop, the frames representing the largest and smallest cross-sectional LV m-mode images were selected as the systolic and respective diastolic phases of the heart. The mice underwent M-mode echocardiography using Vivid 7 Dimension (GE Healthcare, WI) equipment [8]. The standard echocardiogram included assessment of LV Ejection fraction (EF), Fractional Shortening (FS), LV myocardial mass and internal diameters at systole and diastole. The LV internal diameters were traced and measurements were carried out with equipment-inbuilt software.

#### Combined miRNA/total RNA isolation, miRNA profiling and reverse transcription

Total RNA including miRNA was isolated from ventricular tissue or HL-1 cells using mirVana™ miRNA isolation kit, according to the manufacturers protocol. Quality assessment of the total RNA was performed using Agilent 2100 Bioanalyzer. Prior to hybridization total RNA (8 µg) was labeled with biotin which was hybridized to LNA-modified capture probes coupled to beads. This was then bound to a streptavidin-phycoerythrin conjugate reporter molecule. Comprehensive profiling of all the mouse/rat miRs annotated in the miRBase 8.0 was performed using the FlexmiR™ MicroRNA Labeling Kit. Subsequent washing, hybridization and analysis of samples were performed according to the manufacturer. The final reading was obtained from a Luminex 200 analyzer and the Luminex IS Software Version 2.3. Data Normalization and data analyses were done using the software FlexMax_Beta_A02 [1].29 and statistical significance was calculated using Student’s *t* test. Specific Taqman assays for miRNA and mirVana qRT-PCR miRNA RT Kit were used with real-time PCR system and Taqman universal master mix. Levels of miRNA were quantified with the relative quantification method using snoRNA202 as the housekeeping miRNA. The transcription levels of the genes and housekeeping controls were detected using SYBR green-I and expression levels of miRNA and mRNA were quantified employing the 2(−ΔΔct) relative quantification method.

#### Measurement of in vivo tissue redox status with localized Electron Paramagnetic Resonance (EPR)

In vivo tissue redox status was determined by EPR as described previously with modifications [9]. Briefly, 2,2,5,5-tetramethyl-3-carboxylpyrrolidine-*N*oxyl (PCA) was employed as the spin probe to generate a three-line EPR spectrum. The nitroxide solutions were prepared in PBS and kept frozen until use. PCA solution (10 mM, 20 µl) was intramuscularly injected into the left ventricular region. EPR spectra were acquired with a surface loop resonator placed on top of the heart. The lower field peak-height was monitored with time to determine the rate of probe reduction.

#### TBARS assay

As a marker of lipid peroxidation in the ventricular myocardial tissue, malondialdehyde (MDA) levels were detected by the thiobarbituric acid reactive substances (TBARS) method. TBARS assay was performed as reported previously [4] with some modifications. 25–50 mgs of myocardial tissue was ground in liquid N2 and was homogenized in methanol: Butylated hydroxytoluene (950 ul:50 ul). The homogenate was mixed with 1 ml PBS (pH 7.4) and 2 ml chloroform and vortexed for 1 min followed by sonication for 1 min and centrifugation for 5 min at 14000 rpm. The chloroform layer was collected in glass tubes and evaporated under nitrogen gas. 400 ul sodium dodecyl sulfate and 500 ul of thiobarbituric acid reagent was added and boiled for 1 hour at 95°C with glass marbles on the top of the tubes to avoid evaporation. Tubes were then removed and cooled on ice for 10 mins. They were then centrifuged at 3000 rpm for 15 minutes. The supernatant was removed and absorbance was read at 532 nm. Values were compared with MDA standards to determine the degree of lipid peroxidation in the tissue.

#### Reduced (GSH) and oxidized (GSSG) glutathione assay

GSH and GSSG were detected in the ventricular myocardial tissues using an HPLC coulometric electrode array detector (CoulArray Detector, model 5600 with 12 channels; ESA Inc., Chelmsford, MA, USA) as described previously [10], [11], [12]. The CoulArray detector employs multiple channels set at specific redox potentials. Data were collected using channels set at 600, 700, and 800 mV. Samples were snap frozen and stored in liquid nitrogen until HPLC assay. Sample preparation, composition of the mobile phase, and specification of the column used were as previously reported [12], [13].

#### Lactate measurement

Mice were euthanized; hearts were dissected out and the ventricular tissue was homogenized in the assay buffer provided with the Lactate Assay Kit (Biovision). Assay for lactate was done according to the protocol provided with the kit.

#### Preparation and incubation of mitochondria and measurement of respiration

Mitochondria extraction was done and respiration rates of cardiac mitochondria were determined immediately following isolation using a Clark oxygen electrode and an oxygen monitor (Yellow Springs Instrument, Yellow Springs, OH). Mitochondria was isolated as published previously [9]Mice were euthanized and the heart was dissected out and immediately placed in ice cold buffer (220 mM mannitol, 70 mM sucrose, 50 mM Tris-HCl, 10 mM EDTA, 50 mM KCl and 2 mgs/ml BSA, pH 7.4). After removal of extra ventricular tissue, the hearts were weighed and then two hearts were pooled together. They were sliced into very small pieces using a scissor and then finely minced using a polytron (5 sec bursts) in a volume of buffer 10 times the combined weight of the two hearts. Homogenization was accomplished using a Dounce homogenizer by two strokes of pestle A (larger), followed by three strokes of pestle B (smaller). After two washes, the final pellet was resuspended in buffer (250 mM Sucrose, 50 mM Tris-HCl, 3 mM EGTA, pH 7.4). All steps were performed at 0–4°C and without delay to minimize the potential for degradation during the isolation procedure. Mitochondrial protein concentration was determined spectrophotometrically using the Bradford assay. Mitochondrial respiration was measured as published previously [14]. All measurements were completed within 1 h. Succinate (10 mM) was used as substrate. State 3 respiration rates (ADP-dependent oxygen consumption) were determined following the addition of 200 µM ADP, whereas the rates measured following the consumption of ADP as it was converted to ATP were taken as the state 4 respiration rates (ADP-independent oxygen consumption). Respiratory Control Ratio (RCR) was calculated as a ratio of State 3 to State 4 respiration.

#### Cell culture

Murine HL1 cardiomyocytes [15] were cultured in flasks coated with 12.5 µg/ml fibronectin and 0.02% gelatin maintained in Complete Claycomb Medium supplemented with 100 µM norepinephrine (consisting of 10 mM Norepinephrine dissolved in 0.3 mM L-ascorbic acid), 4 mM L-glutamine, 10% fetal calf serum, 100 units/ml penicillin and 100 µg/ml streptomycin at 37°C in a humidified atmosphere of 95% air and 5% CO_2_.

#### miRNA delivery to cells

Transfection of HL-1 cells was performed as described [16]. Briefly, HL-1 cells (0.15×10^6^ cells per well in 12-well plate) were seeded in antibiotic-free supplemented Claycomb medium 24 h before transfection. DharmaFECT 1 transfection reagent was used to transfect cells with hsa-miRNA-15b mimic **(Supplementary Fig. S3)** as per the manufacturer’s instructions. Transfection of miRNA mimic negative controls was performed for the control groups. Cells were harvested after 72 h of such treatment.

#### Dicer knockdown in cells

si-Dicer was used to knock down Dicer in cells using a standard transfection protocol. Reagents were obtained from Thermo Scientific **(Supplementary Fig. S3)**.

#### Measurement of mitochondrial membrane potential (**Δ**Ψ)

*JC-1 assay* Mitochondrial membrane potential changes were assessed using the lipophilic cationic dye JC-1 as reported previously [17]. Results (percentage of negative cells) were normalized to either non-transfected cells or cells transfected with the scrambled mimic control while CCCP treated cells were used as a positive control. *TMRM assay* Mitochondrial membrane ΔΨ was measured using the fluorescent lipophilic cationic dye TMRM, which accumulates within mitochondria in a potential dependent manner [18]. HL1 cells transfected with mir-15b mimic and after 72 h were reseeded on glass coverslips. Following 24 h of re-seeding, the cells were stained with 8 nM TMRM and 0.5 ml/ml plasma membrane potential indicator (PMPI) for 30 min at 37°C in the dark. Cells were washed with PBS, and digital images of stained live cells were collected using a Zeiss Axiovert 200 M microscope [19].

#### Measurement of in vivo mitochondrial membrane potential using TMRM staining

In vivo measurement of mitochondrial membrane potential was assessed using TMRM staining. Frozen sections were incubated with 50 nM TMRM solution in PBS for 15 minutes and red fluorescence was quantified under the microscope [20].

#### Anti-miRNA-15b delivery

Mice were injected with tamoxifen or corn oil as described previously. LNA-modified anti-miRNA-15b was administered through the tail vein at a dose of 80 mg/kg, 24 h prior to the first tamoxifen/corn oil injection. This was followed by three more injections at a dose of 40 mg/kg at 0 h, 24 h and 72 h after the first tamoxifen/corn oil injection. Echo and MRI imaging was performed on day 7 and the hearts were collected for RNA and protein analyses.

Mice were injected with tamoxifen or corn oil as described previously. LNA-modified anti-miRNA-15b was administered through the tail vein at a dose of 80 mg/kg, 24 h prior to the first tamoxifen/corn oil injection. This was followed by three more injections at a dose of 40 mg/kg at 0 h, 24 h and 72 h after the first tamoxifen/corn oil injection. Echo and MRI imaging was performed on day 7 and the hearts were collected for RNA and protein analyses. Saline or a standard LNA™ microRNA inhibitor negative control was used as controls.

#### Western blots

Western blot was performed with primary antibodies against Dicer (1∶400), Pim1 (1∶500) and GAPDH (1∶10,000).

#### Laser Microdissection and Pressure Catapulting (LMPC)

Mouse heart samples (ventricles) frozen in optimum cutting temperature compound were cut into 10 µm-thick sections using a cryotome. The sections were placed on PEN-covered glass slides. and stained with hematoxylin to identify the cardiomyocytes as per method published by us before [21], [22]. LMPC was performed using a PALM MicroLaser system (PALM-Zeiss, Bernreid, Germany) containing a PALM MicroBeam (driven by PALM MicroBeam software) and a PALM RoboStage and, for high-throughput sample collection, a PALM RoboMover (driven by PALM RoboSoftware version 2.2). A typical setting used for laser cutting was a beam size of 30 µm and laser strength of 30 mV under a×10 ocular lens. Mouse heart sections were placed on PEN membrane-covered glass slides (PALM Industries) that had been treated with RNAZap (Ambion, San Antonio, TX) as instructed by the manufacturer. Tissue areas of approximate size of 4×10^6^ µm^2^ containing cardiomyocytes (approx. 1500 cells) were cut under a×10 ocular lens and catapulted directly into 25 µl of an RNA lysis solution.

#### Immunohistochemistry

Formalin-fixed paraffin-embedded or OCT-embedded frozen specimens were sectioned and immunohistochemical staining of sections were performed. Image analysis software (Axiovision 4.3, Zeiss, Germany) was used to quantify fluorescence intensity (fluorescent pixels) and analyzed as a percent change in relative fluorescence unit (RFU).

#### miRNA target reporter luciferase assay

HL1 cells were transfected with 100 ng of pLuc-Pim1–3′UTR plasmid (Signosis) using Lipofectamine LTX/Plus reagent according to the manufacturer's protocol. Normalization was achieved by co-transfection with *Renilla* plasmid (10 ng). Cells were lysed after 48 h, and luciferase activity was determined using the dual-luciferase reporter assay system. Data are presented as ratio of firefly to *Renilla* luciferase activity.

#### Statistical analyses

Data are reported as mean ± S.D. of at least three independent experiments as indicated in respective figure legends. Difference between means was tested by Student’s *t*-test. Comparisons between multiple groups were made by analysis of variance. *p*<0.05 was considered statistically significant.

#### Ethics in animal research

For all animal work, approval was granted by Ohio State University's Institutional Animal Care and Use Committee (IACUC)and the investigation conforms to the Guide for the Care and Use of Laboratory Animals published by the United States National Institutes of Health. For euthanasia, animals were placed inside a closed chamber with a CO2 source tube connected to the chamber. CO2 gas was then be pumped into the chamber in a way to minimize stress to the animal until the animal was no longer breathing or showed signs of life. The animal was then removed and the neck was cervically dislocated to ensure death. This procedure was done within a ventilated hood.

## Results and Discussion

### *Dicer* Deletion in the Adult Heart Leads to Rapid Loss of Cardiac Function

**Figure 1:**
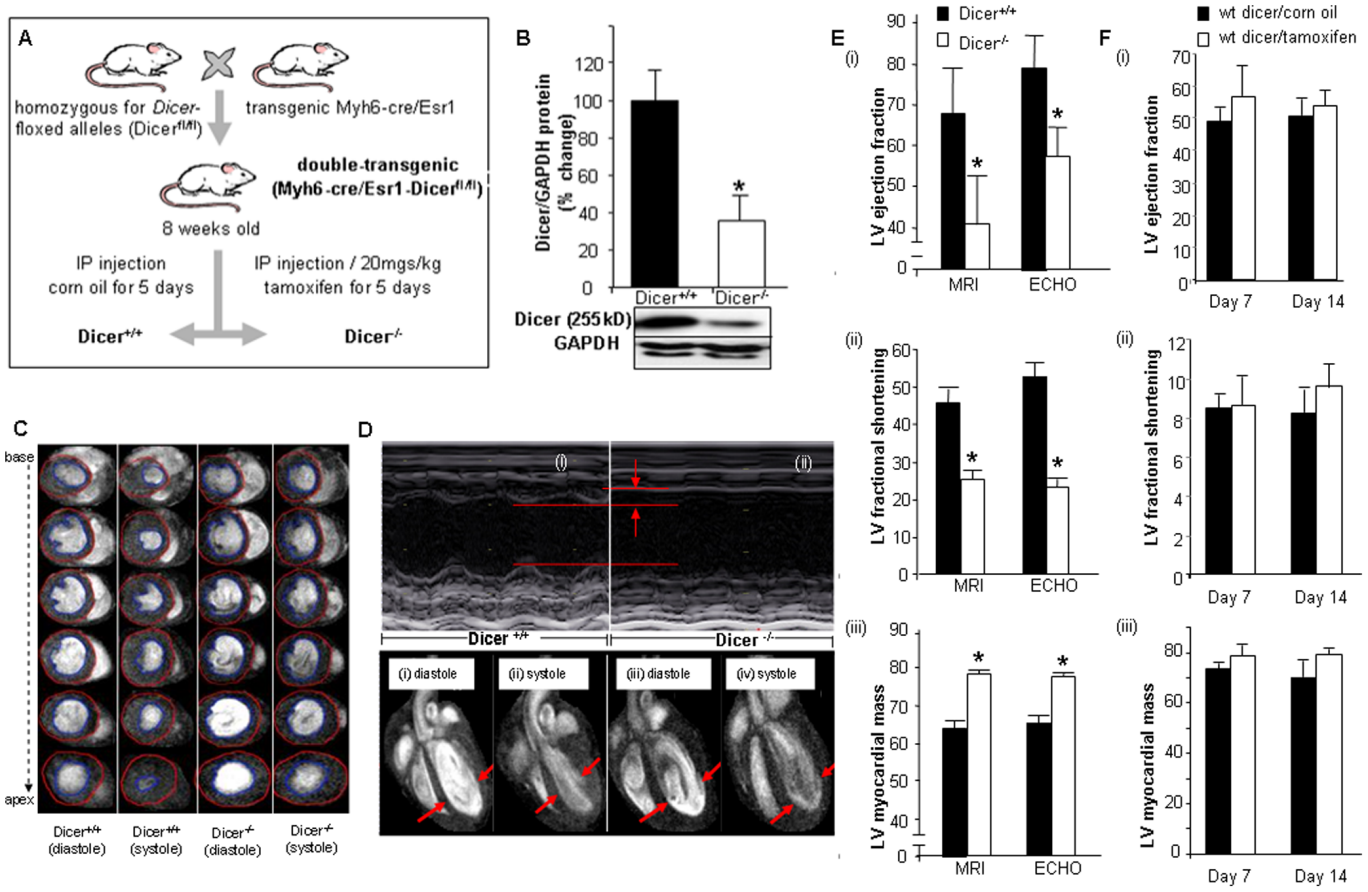
Cardiac specific Dicer deletion leads to cardiac hypertrophy and impaired cardiac function. (**A**) Generation of Myh6-cre/Esr1-Dicer^fl/fl^ mice. (**B**) Western Blot showing reduced Dicer expression following tamoxifen injection. (**C**) 11.7T images of short axis at diastole and systole of 1.0 mm slices stacked along columns from base to apex of the left ventricle of both Dicer^+/+^ and Dicer^−/−^ mice. Blue contour lines indicate LV volume. (**D**) Representative M-mode images of Dicer^+/+^ and Dicer^−/−^ mice. The data indicates decreased contractility and increased left ventricular (LV) internal dimensions in Dicer^−/−^ mice. 11.7T MRI images of long axis at diastole (i & iii), systole (ii & iv) cardiac 11.7T MRI images in Dicer^+/+^ and Dicer^−/−^ mice. The diastole of Dicer^−/−^ mice was enlarged due to insufficient contraction of the heart (shown by arrows). (**E**) Quantification of LV fractional shortening, LV myocardial mass, and LV ejection fraction (EF) by MRI and ECHO. Solid bars represent Dicer^+/+^ and open bars represent Dicer^−/−^. (**F**) (i) Ejection fraction, (ii) fractional shortening and (iii) LV mass in wild type corn oil or tamoxifen injected mice.

Dicer deficiency is emerging as a key factor determining health outcomes in humans. Low Dicer expression has been reported in heart failure patients [4]. However, very little is known about the underlying mechanisms. To dissect the significance of Dicer in mammalian biology, several groups have disrupted the *Dicer* gene in different organs in mice [23], [24].http://circres.ahajournals.org/cgi/content/full/100/8/1164 - R25-M150524#R25-M150524 Cardiac-specific deletion of *Dicer* leads to defects in heart development and embryonic lethality [1], [4], demonstrating that Dicer is necessary for murine development. In order to study the effects of Dicer depletion in adult heart, we developed a double transgenic Myh6-cre/Esr1-Dicer^fl/fl^ mice by breeding mice with a floxed *Dicer* allele [25] with mice expressing a tamoxifen-inducible Cre recombinase protein, flanked on each end with a mutated murine estrogen receptor ligand binding domain, under the control of the cardiac-specific murine alpha-myosin heavy chain (Myh6) promoter. Intraperitoneal tamoxifen injection (once daily, 5 consecutive days) activated the Cre recombinase resulting in *Dicer* null mice (Dicer^−/−^). The Myh6-Cre mice were obtained from Jackson Laboratories and efficacy of the Cre driver is available on their website (The Myh6-cre mice were obtained from Jackson Laboratories. Information regarding the efficacy of the Cre-driver is available on their website (http://cre.jax.org/Myh6-creEsr1/Myh6-creEsr1.html) and also characterized in literature [25]. The control set (Dicer^+/+^) was generated by injecting littermates with equivalent amount of vehicle (ethanol/corn oil) **(** **Fig. 1A** **)**. Within 7 days from the start of tamoxifen delivery, Dicer protein was reduced by 65% **(** **Fig. 1B** **)**. Long and short axis serial 11.7T MRI images and M-mode echocardiography demonstrated **(** **Fig. 1C–D** **and Supplementary Movie S1 and S2)**. Objective assessment of the MRI and echocardiography data consistently demonstrated significant (*p*<0.05, n = 5) decline in cardiac function as manifested by decreased fractional shortening, lower ejection fraction and increased left ventricular myocardial mass **(** **Fig. 1E** **)** and left ventricular internal diameter **(Supplementary Fig. S1).** No change in wall thickness was observed. These were not manifested in wild type mice injected with equal dosage of tamoxifen **(** **Fig. 1F** **)**.

Along with induction of hypertrophy and functional defects, we also observed myofiber disarray and initial signs of onset of fibrosis **(Supplementary Fig. S2)** upon loss of Dicer in the adult murine myocardium and is consistent with other studies reported while this work was in progress. [3].

### *Dicer* Deletion in the Adult Heart Leads to Oxidative Stress and Mitochondrial Dysfunction

**Figure 2:**
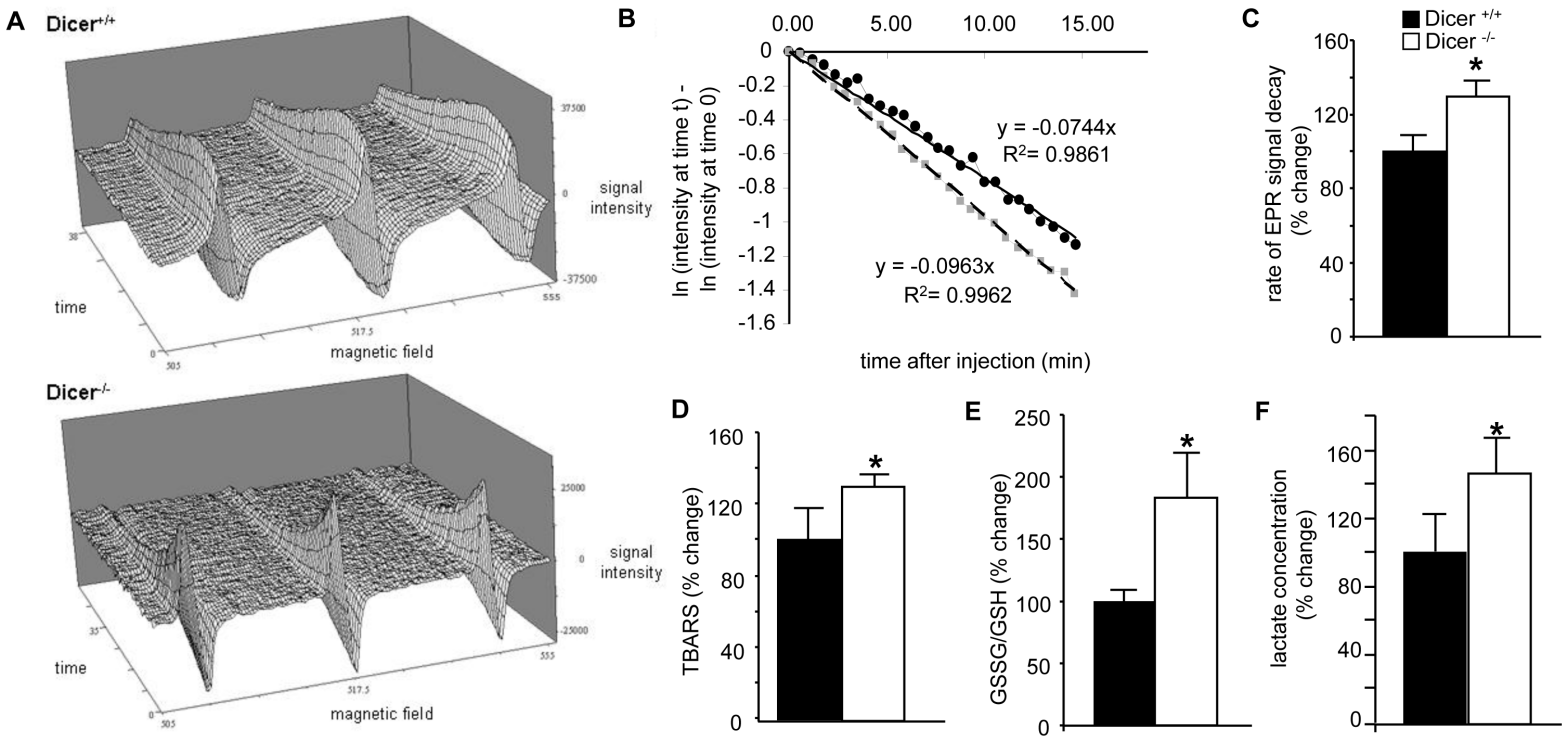
Dicer deletion in the adult heart leads to oxidative stress. (**A**) Representative EPR imaging of nitroxide radical decay in (A) Dicer^+/+^ and (B) Dicer^−/−^ hearts. (**B**) Time course of average % signal change in the region of interest (ROI). Logarithmic values of signal change (normalized to the initial signal at time = 0) in the ROIs are plotted with respect to time. Decay rate constants were obtained from the slope of linear decay after peak. Line with black circles represent Dicer^+/+^ and line with grey circles represent Dicer^−/−^. (**C**) Bar-graph showing the measured rate constants of nitroxide reduction in the tissues. (**D**) Thiobarbituric acid–reactive substances (TBARS), an indicator of lipid peroxidation was measured from Dicer^+/+^ and Dicer^−/−^ hearts and was significantly higher in the later. (**E**) Total GSSG to GSH ratio in Dicer^+/+^ mice heart compared to Dicer^−/−^ heart. (**F**) Lactate levels measured in Dicer^+/+^ and Dicer^−/−^ mice hearts.

**Figure 3:**
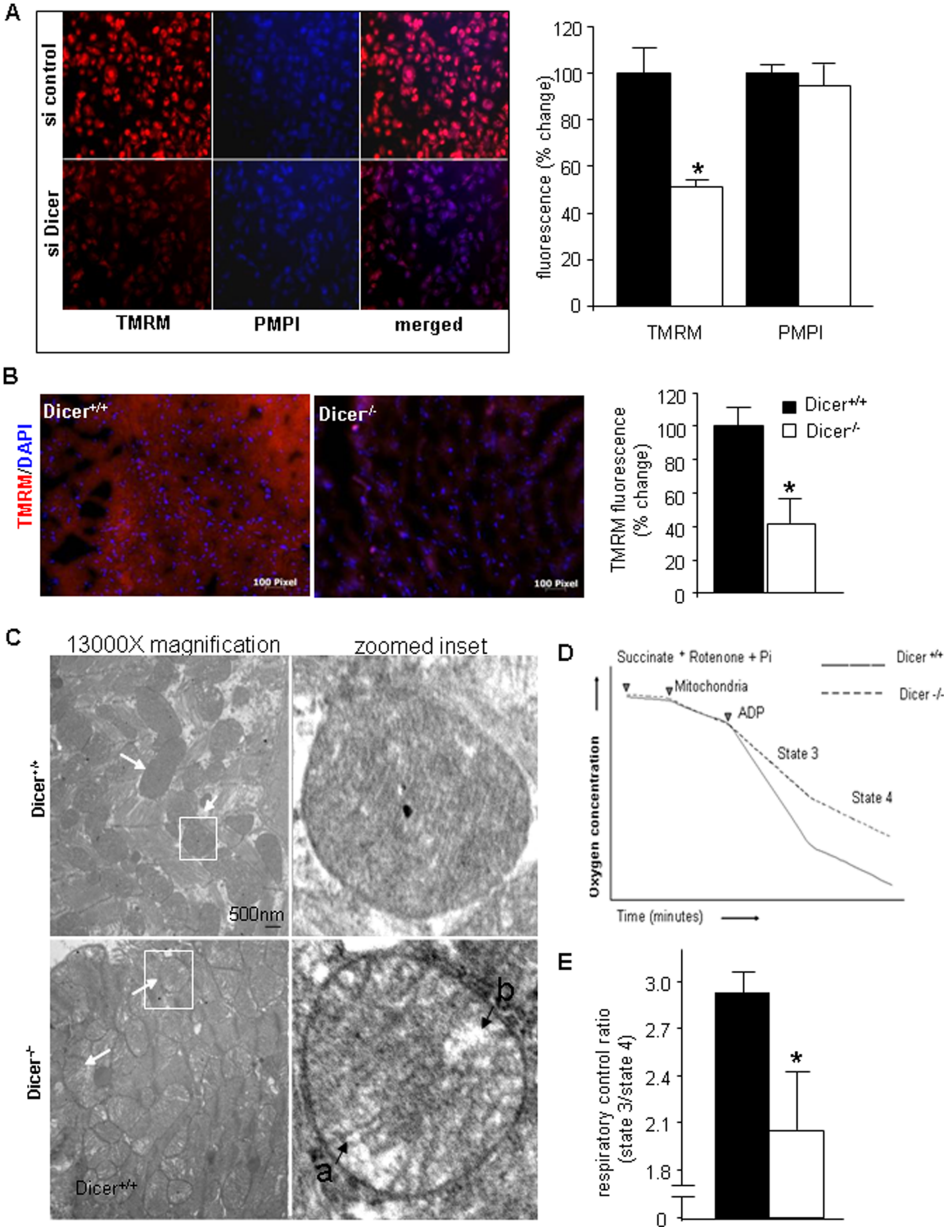
Dicer deletion in the adult heart leads to mitochondrial dysfunction. (**A**) TMRM assay in HL-1 cells transfected with si-control and si-Dicer. (**B**) TMRM assay on Dicer^+/+^ and Dicer^−/−^ heart sections. Bar graph quantifying TMRM fluorescence demonstrates lower TMRM fluorescence in Dicer^−/−^ hearts (n = 3). (**C**) Representative TEM images of mitochondria from heart tissue. Arrows indicate mitochondria. Single mitochondria in white boxes have been zoomed into in the right panel. Labels *a)* indicate increased inter-cristae space as compared to the mitochondria in the Dicer^+/+^ heart and *b)* points to loss of integrity of the cristae structure. (**D**) Representative diagram showing experiment for RCR measurements from isolated mitochondria. (**E**) Respiratory coupling ratio (RCR) was significantly lower in Dicer^−/−^ mice indicating mitochondrial dysfunction.

Hypertrophy and cardiac failure has often been associated with mitochondrial dysfunction and oxidative stress [26], [27], [28]. Mitochondrial energy deficiency is known to underlie dilated cardiomyopathy characterized by early mechanical dysfunction followed by a decline in left ventricular systolic function [29]. We therefore wanted to determine the redox and mitochondrial health of the Dicer depleted hearts. Redox state of the *Dicer* deleted adult heart was determined by EPR spectroscopy. After intramuscular injection of 2, 2, 5, 5,-tetramethylpyrrolidine-1-oxyl-3-carboxylic acid (PCA) to the heart, decay of nitroxyl radicals was studied. A faster decay in *Dicer* deleted hearts was seen that has been considered indicative of a higher abundance of reactive oxygen species [9] (**Fig. 2A–C**, *p*<0.05, n = 3). This observation was consistent with elevated lipid peroxidation (**Fig. 2D**; *p*<0.05, n = 3) and glutathione oxidation (**Fig. 2E**; *p*<0.05, n = 4), indices of oxidative stress, in the hearts of Dicer^−/−^ mice. Consistent with this observation demonstrating impairment in oxidative metabolism it was noted that lactate levels in the heart of Dicer^−/−^ mice were significantly higher (**Fig. 2F**; *p*<0.05, n = 4). Overt oxidative stress in Dicer-deficient hearts evident in this study pointed towards underlying mitochondrial dysfunction [28], [30]. In order to further elucidate the cause and effect relationship, we used beating HL-1 cells which have been demonstrated be physiologically and functionally similar to adult cardiomyocytes [15], [31]. Knocking down Dicer in HL-1 cells using a siRNA compromised mitochondrial membrane potential (determined by TMRM fluorescence) **(** **Fig. 3A** **)**. To further confirm the in-vitro findings, we performed in-vivo TMRM staining on Dicer^+/+^ and Dicer^−/−^ heart sections and observed similar results **(** **Fig. 3B** **)**. We analyzed mitochondrial structure by transmission electron microscope and observed a loss of mitochondrial structural integrity in cardiac tissue from Dicer^−/−^ mice (**Fig. 3C**). Indeed, Dicer deficient cardiac tissue showed evidence of matrix swelling represented by greater inter-cristae space and sometimes unfolded cristae localized at one pole of the organelle as compared to intact cristae uniformly distributed across the organelle in Dicer^+/+^ tissue. This contention was supported by analysis of respiration of isolated heart mitochondria which led to the observation that the respiratory control ratio (RCR) was significantly decreased (*p*<0.05, n = 5) in cardiac mitochondria from Dicer^−/−^ mice compared with Dicer^+/+^ animals (**Fig. 3D–E**). Taken together, these findings demonstrate that *Dicer* deletion induced loss of cardiac function of the adult heart is associated with mitochondrial dysfunction and oxidative stress.

### Cardiac Specific *Dicer* Deletion Leads to Early Onset Changes in Specific miRs

**Figure 4:**
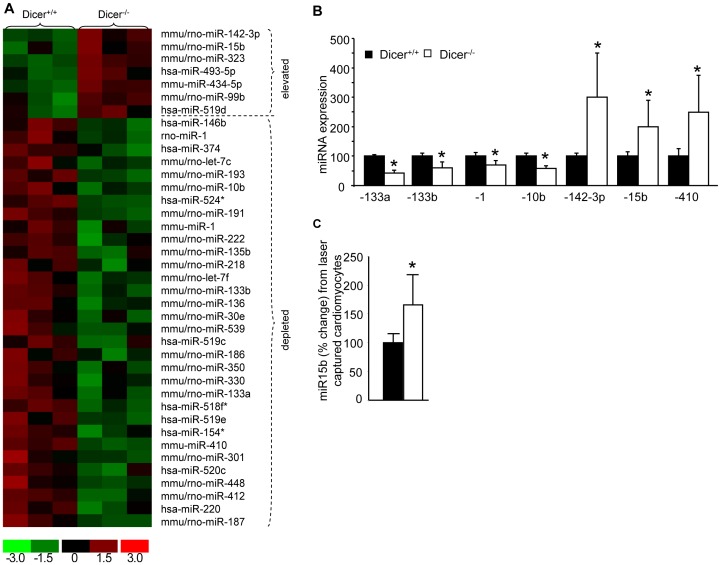
Cardiac specific *Dicer* deletion leads to early onset changes in some miRNAs including miRNA-15b. (**A**) Heat map generated for the differentially expressed miRNAs in Dicer^+/+^ and Dicer^−/−^ mice. (**B**)Validation of selected miRNAs by real-time PCR. (**C**) miR-15b expression in laser captured cardiomyocytes from Dicer^+/+^ and Dicer^−/−^ tissues.

Dicer supports microRNA biogenesis. The observation that *Dicer*-deletion in the adult heart caused early onset mitochondrial dysfunction led to the search for miRNA-dependent pathways targeting mitochondrial function in the heart. The current work capitalizes on the observation that the onset of cardiac pathologies and dysfunction after conditional *Dicer* deletion in adult hearts is rapid, such that overt functional deficiencies are manifested in the first week. miRNA profiling was performed at such early time-point in order to identify the primary factors underlying cardiac dysfunction following *Dicer* deletion. We identified 39 miRNAs (out of 327 listed in miRbase 8.0) which significantly changed (*p*<0.05, n = 3) after the first week of tamoxifen injection **(** **Fig. 4A** **)**. Of the 39 candidate miRNAs identified, 7 were elevated while 32 were depleted. Results were confirmed using QPCR **(** **Fig. 4B** **)**.

A possible explanation for such a few being significantly changed can be explained by our study design. First, our approach led to 65% reduction in Dicer activity. The residual Dicer function is likely to prevent rapid depletion of all miRNAs and the model from being more toxic. Second, to identify the key causative factors underlying cardiac dysfunction following *Dicer* deletion the study design was focused on capturing miRNAs at an early time point, when only a few miRNAs were significantly changed, thus avoiding a more toxic effect, but enough to observe the effects. Additionally, as the literature will support [3], [4], [32], tissue miRs have been reported to be both up regulated and down-regulated in the short term following *Dicer* deletion. A possible explanation for the up-regulation of miRs may be as follows: **(i)** certain miRs have a much longer half-life as compared to others [33], [34], [35], [36], [37], [38]; **(ii)** because Cre is expressed only in heart muscle (MHC-MerCreMer), Dicer will be depleted only in the cardiomyocytes following tamoxifen treatment. As suggested previously [3], it is possible [39], [40], [41] that miRs upregulated in non-myocyte cells may get transported across cells and cause gene silencing in myocytes [39], [40], [41]. Further studies are required to elucidate the mechanism behind this phenomenon and is beyond the scope of this paper.

### Elevated miRNA-15b Silences Pim-1 Kinase

**Figure 5:**
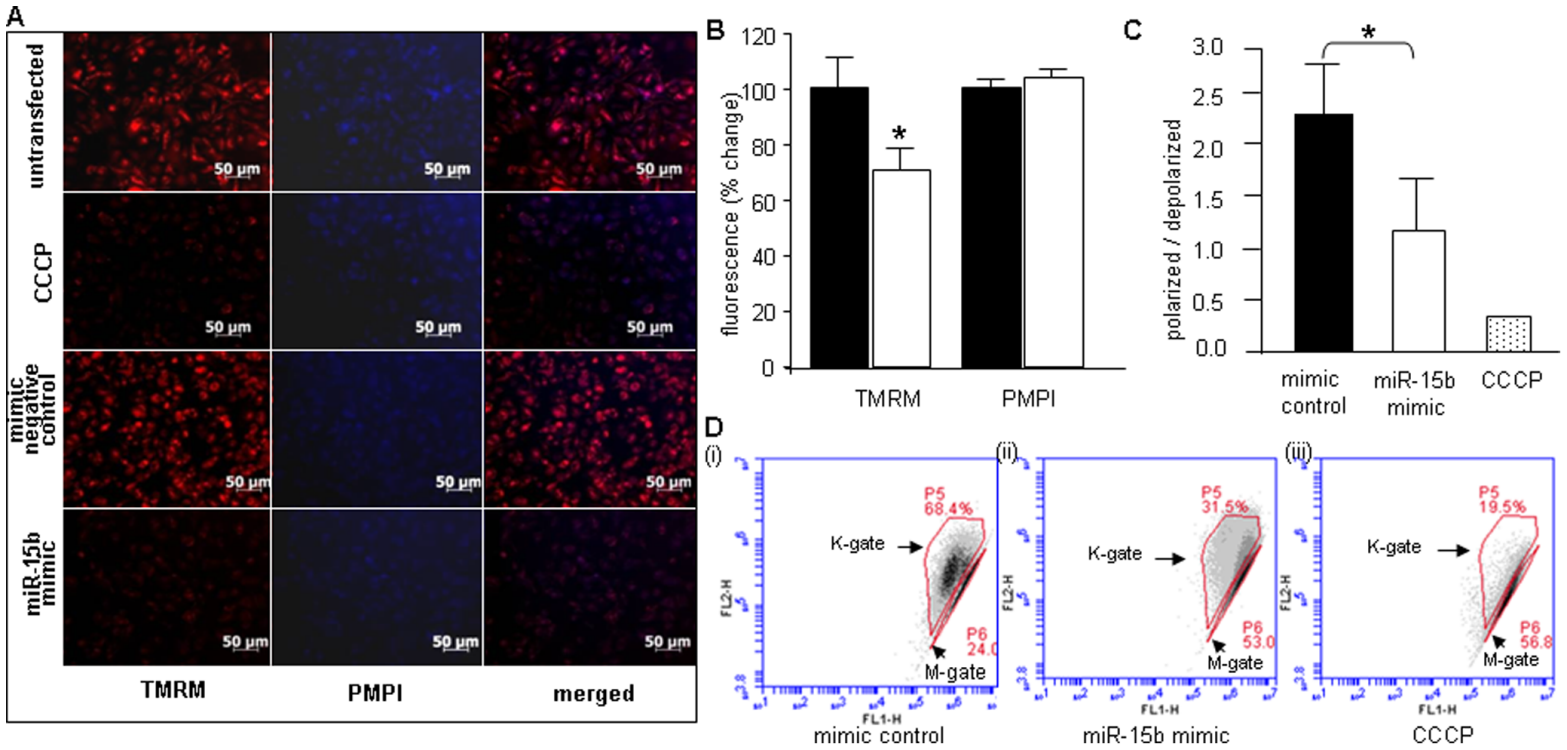
miR-15b over expression in HL-1 cells compromises mitochondrial membrane potential. (**A**,**B**) HL-1 cells were transfected (72 hours) with mimic negative control or miRNA-15b mimic. Untransfected cells were used as negative control and cells transfected with carbonyl cyanide m-chlorophenylhydrazone (CCCP) were used as positive control (left panel –8 nM tetramethylrhodamine methyl ester (TMRM); center panel - 0.5 µl/ml plasma membrane potential indicator (PMPI); right panel - merged image). Bar graph shows significant decrease in TMRM with no change in PMPI. Solid bars represent mimic negative control while open bars represent miRNA-15b mimic. (**C**,**D**) Loss of mitochondrial membrane potential in HL-1 cells transfected with miRNA-15b mimic, as assessed by JC-1 flow cytometry 72 h post-transfection. Cells were transfected with a (i) mimic control, (ii) miRNA-15b mimic or (iii) treated with CCCP Arrows (K gate) indicate cells containing JC-1 aggregates resulting from intact mitochondria; M gate indicates cells with low or collapsed mitochondrial membrane potential. Ratio of polarized to depolarized cells calculated as a ratio between cells in K gate to M gate. Data indicate decrease in membrane potential upon up-regulation of miRNA-15b. (n = 3).

**Figure 6:**
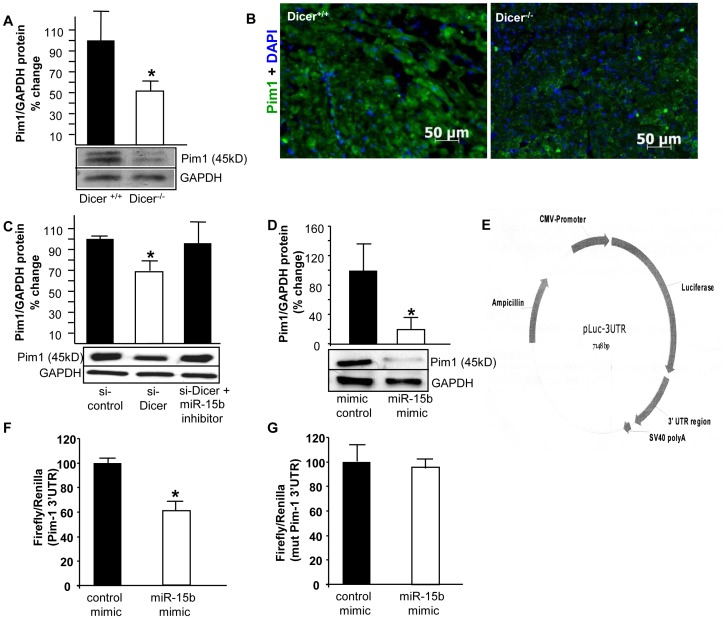
Pim-1 is a target of miRNA-15b and protein expression is reduced in Dicer^−/−^ mice. (**A**) Western blot analysis of Pim-1 protein from whole tissue homogenates from Dicer^+/+^ and Dicer^−/−^ mice hearts. (**B**) Immunohistochemistry showing reduced Pim-1 expression in Dicer^−/−^ cardiac tissue. (**C**) Western blot analysis of Pim-1 protein from HL-1 cells transfected with si-control, si-Dicer and si-Dicer with miR-15b inhibitor. Quantification of Pim-1 expression was performed using densitometry. (**D**) Western blot analysis of Pim-1 protein from HL-1 cells transfected with scrambled control and miRNA-15b mimic, (**E**) Plasmid construct for pLuc-Pim1–3′UTR plasmid. (**F**) miRNA target reporter luciferase assay with Pim-1 3′UTR. Results were normalized with data obtained from an assay with *Renilla* luciferase. (**G**) Luciferase activity in HL-1 cells transfected with a Pim1-mutated 3′UTR and miR-15b mimic.

We screened for miRNAs targeting mitochondrial function among the significantly differentially expressed miRNAs, using publicly available prediction softwares like TargetScan™ v 5.1 and Pictar™. In our search, elevated miRNA-15b emerged as a key candidate as it was computationally predicted to silence some key proteins like Pim-1 kinase, ESRRG, Mfn-2 which are required for preserving proper mitochondrial function and integrity. We used laser capture microdissection [22], [42], [43], [44] to confirm that miR-15b was indeed elevated in cardiomyocytes. We recognize that the other differentially expressed miRs may also play a significant role in development of the pathology in *Dicer* deleted hearts and needs to be investigated as a future work. Our study was directed to Pim-1 kinase, silencing of which is known to directly cause cardiac dysfunction. Pim-1 is a crucial requirement for downstream cardio protective effects of Akt signaling [45]. Increase in Akt abundance and phosphorylation after pathological injury by infarction or pressure overload does not protect the myocardium in Pim-1-deficient mice [45]. Loss of Pim-1 also prevents the long term structural and functional benefits obtained from cardiac progenitor cell based therapies indicating its importance in myocardial repair and regeneration [46], [47]. Transgenic over-expression of Pim-1 in the myocardium protects mice against infarction [45]. Specifically, Pim-1 kinase is required for preserving mitochondrial integrity in cardiomyocytes [48], and also blunts cardiac hypertrophy [49]. Pim-1 kinase over-expression also reduces calcium mediated mitochondrial swelling, *t*-bid-induced cytochrome-C release and peroxide-induced membrane depolarization [50]. Pim-1 inhibits apoptosis-related mitochondrial dysfunction [51] and Pim-1 inhibition results in apoptosis and is closely related to the decrease in Akt and Bad phosphorylation and increase in cleaved caspase-3/−9 activities [52]. Delivery of miR-15b to HL-1 cells using a mimic compromised mitochondrial membrane potential (TMRM fluorescence) similar to what was observed upon Dicer knockdown **(** **Fig. 5A,B** **)**. Mitochondrial membrane potential changes were also assessed using the JC-1 assay which demonstrated that over expression of miRNA-15b in HL-1 cells compromised ΔΨ **(** **Fig. 5C,D** **)**. Pim-1 kinase protein level was observed to be low in heart tissue isolated from Dicer^−/−^ mice by western blot (**Fig. 6A**, *p*<0.05, n = 6) and immunohistochemistry (**Fig. 6B**). HL-1 cells transfected with si-Dicer showed lower Pim-1 protein expression. This effect was ameliorated when cells were co-transfected with a miRNA-15b inhibitor pointing towards a direct role of miRNA-15b in silencing Pim-1 (**Fig. 6C**, *p*<0.05, n = 3). In order to validate whether miRNA-15b silences Pim-1 kinase, HL-1 cells were transfected with miRNA-15b mimic and lowered Pim-1 kinase protein levels was observed (**Fig. 6D**; *p*<0.05, n = 6). To validate the direct binding between miRNA-15b and the Pim-1 3′ UTR region, miRNA target reporter luciferase assay was performed using the pLuc-Pim1–3′UTR plasmid (**Fig. 6E**) in HL-1 cells. 40% reduction of luciferase activity from the pLuc-Pim1–3′UTR plasmid was observed in miRNA-15b mimic transfected HL-1 cells compared with control **(** **Fig. 6F** **)**. No reduction in luciferase activity was observed using a mutated pLuc-Pim1-mutated 3′UTR **(** **Fig. 6G** **)**. Thus, elevated levels of miRNA-15b silence Pim-1 kinase which may be causatively linked to the observed mitochondrial dysfunction in *Dicer* deleted heart of adult mice.

miRNA-15b also silences ADP-ribosylation factor-like 2 (Arl2), thus compromising cellular ATP levels [53] and therefore, miRNA-15b appears to have a multi-pronged damaging effect on mitochondrial integrity. Of interest, high miRNA-15b is known to be associated with most common types of human heart failure [5], [6].

### Suppression of Elevated miRNA-15b Rescued Function of *Dicer*-deleted Adult Heart

**Figure 7:**
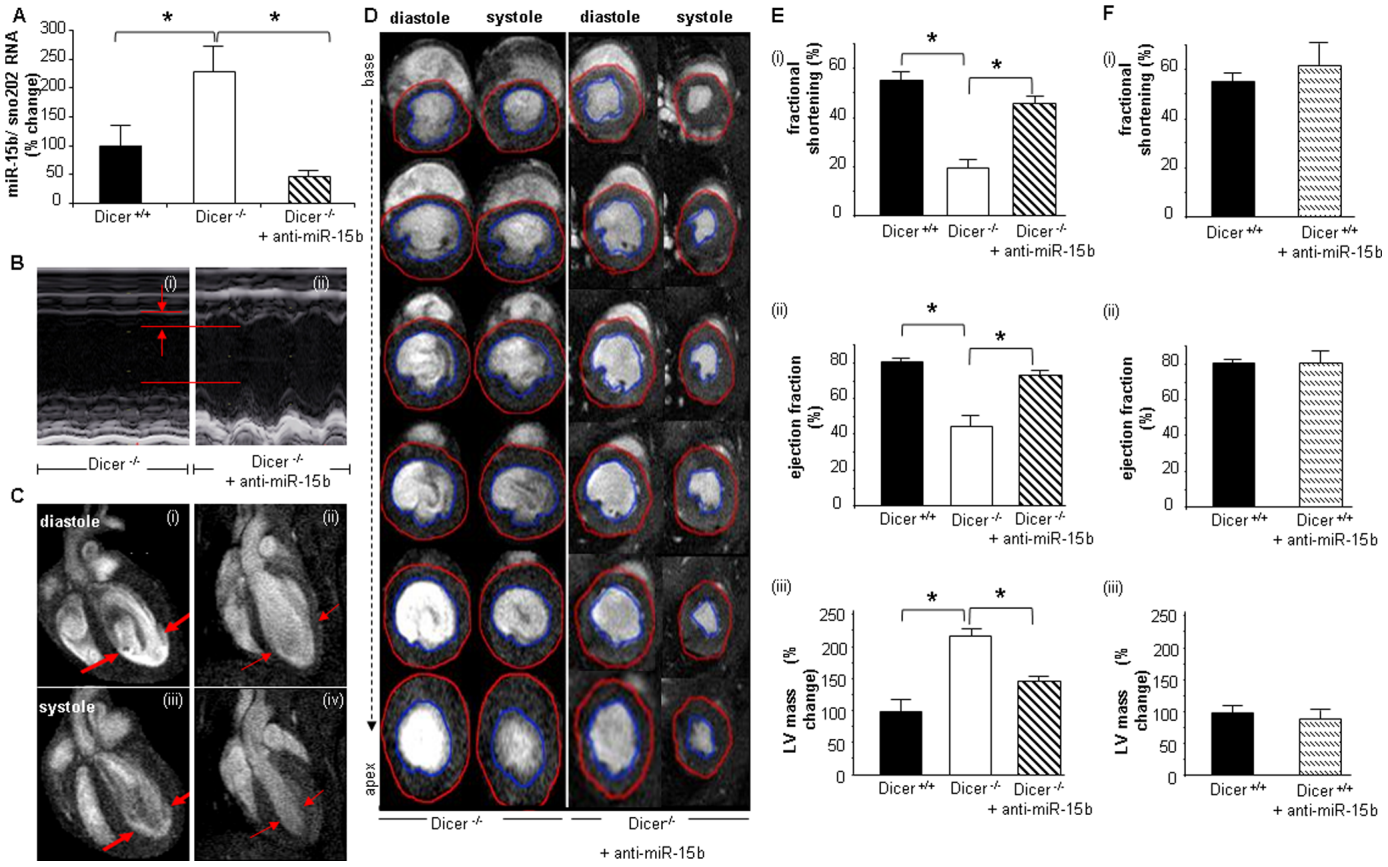
Suppression of induced miRNA-15b prevents impairment of cardiac function. (**A**) Reduced expression of miRNA-15b upon anti-miRNA-15b delivery. (**B)** Representative M-mode images of Dicer^−/−^ and anti-miRNA-15b injected Dicer^−/−^ mice. (**C**) 11.7T MRI images of long axis at diastole (i & ii) and systole (iii & iv) (**D**) 11.7T images of short axis at diastole and systole of 1.0 mm slices stacked along columns from base to apex of the left ventricle as explained in Fig. 1. (**E**) (i) Fractional shortening, (ii) ejection fraction (EF) and (iii) LV mass. (**F**) (i) Fractional shortening, (ii) ejection fraction and (iii) LV mass in saline or anti-miR-15b injected Dicer+/+ (wild type).

**Figure 8:**
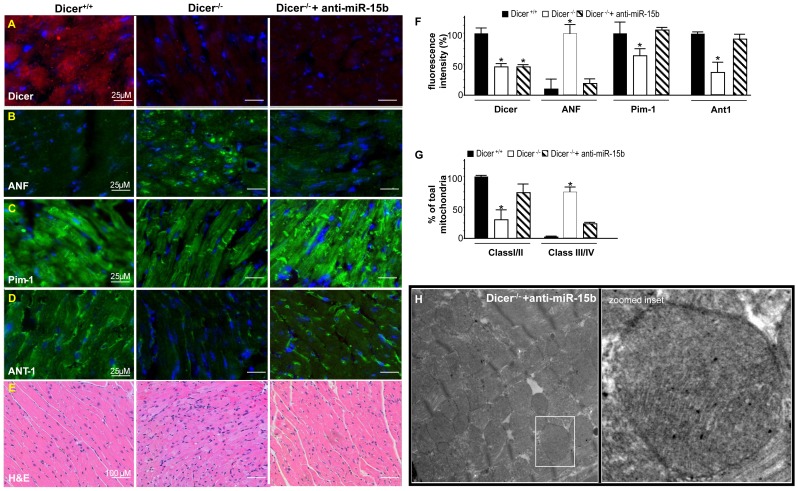
Suppression of induced miRNA-15b attenuates loss of Pim-1 and improves mitochondrial integrity. Immunohistochemical comparison of (**A**) Dicer (**B**) ANF, (**C**) Pim-1(**D**) ANT-1 (**E**) Comparison of cardiac histopathology sections stained with hematoxylin and eosin (H&E) (**F**) Bar graph showing quantification of IHC images. Image analysis software (Axiovision 4.3, Zeiss, Germany) was used to quantify fluorescence intensity (fluorescent pixels) and analyzed as a percent change in relative fluorescence unit (RFU). (n = 3) (**G**) Comparison between % of total mitochondria belonging to Class I/II or Class III/IV. (**H**) Representative TEM images of mitochondria from Dicer depleted heart tissue treated with anti-miR-15b. *Bar graphs represent equal SD on both sides of the mean.*

In order to verify whether, suppression of elevated miRNA-15b, can prevent cardiac dysfunction, we delivered a LNA (locked nucleic acid)-modified anti-miRNA-15b (15mer) through tail vein injection starting one day prior to the beginning of tamoxifen injection. The delivery was successful resulting in significant (*p*<0.05, n = 5) decrease in miRNA-15b levels in *Dicer*-deleted adult hearts (**Fig. 7A**). Cardiac functions were analyzed on day 7 post tamoxifen injection. Anti-miRNA-15b treatment resulted in significant improvement (*p*<0.05, n = 5) in cardiac function as recorded by M-mode echocardiography and long-axis (**Fig. 7B–C** and supplementary movie S1 and S2) as well as short-axis MRI (**Fig. 7D**). In response to anti-miRNA-15b treatment, *Dicer*-deleted adult murine hearts showed improved fractional shortening **(** **Fig. 7E** **-i)**, ejection fraction **(** **Fig. 7E** **-ii)** as well as lower LV mass **(** **Fig. 7E** **-iii)** indicative of attenuated cardiac hypertrophy. No significant change in cardiac parameters were observed upon injecting this anti-miRNA into wild type Dicer^+/+^ mice **(** **Fig. 7F** **)**. Dicer^−/−^ heart exhibited increased ANF expression, a recognized marker of cardiac hypertrophy which wasn’t observed in anti-miRNA-15b treated hearts and was consistent with MRI and Echo results **(** **Fig. 8B,F** **)** Loss of Pim-1 in adult *Dicer*-deleted hearts was attenuated in response to anti-miRNA-15b treatment **(** **Fig. 8C,F** **).** Another mitochondrial marker, ANT-1 [54], was noted to be depleted in the myocardium of Dicer^−/−^ mice while such adverse impact was significantly abrogated in response to anti-miRNA-15b treatment **(** **Fig. 8D,F** **)** supporting a role of elevated miRNA-15b in causing mitochondrial injury. Intact mitochondrial cristae structure was also observed in anti-miR-15b treated Dicer deficient hearts. Total mitochondria was classified as belonging to Class I/II (healthy mitochondria) or Class III/IV (reptured or damaged mitochondria) [55] **(** **Fig. 8G,H** **)**. Hematoxylin/Eosin staining revealed overt histopathological changes such as hypertrophied myofibers and myocyte disarray which were not observed when treated with anti-miRNA-15b **(** **Fig. 8E** **)**. In order to test the specificity of the LNA in the context of elevated miR15b, a standard LNA™ microRNA inhibitor negative control was injected through tail vein and Dicer was knocked out as described above. The negative control could not lower elevated miR-15b levels and all the animals died within 3 weeks of Dicer depletion indicating, no rescue of phenotype **(Supplementary Fig. S4)**.

It needs to be discussed here, that there seems to be a gap between the in-vitro results obtained from HL-1 cells which are of atrial origin and the in-vivo results which relate to ventricular function. Dicer depletion in adult mice displays similar phenotypes in the atria and the ventricles such as atrial enlargement [56]. HL-1 cells are an immortalized cell line and have adult cardiac, morphological, biochemical and electrophysiological properties, including contraction and biochemical response [15]. Cultured HL-1 cells express α-cardiac actin similar to adult mouse ventricle cells [15]. MicroRNA expression profile in HL-1 cells also has clear similarities to that of cardiac left ventricle and no difference was found in miR-15b expression [57]. Because of these attributes, even though HL-1 cells were originally derived from atrial myocytes, they have proven to be useful as a general model for studying contracting (working) cardiomyocytes because of their organized structure and ability to contract in culture [31]. They have been used as a cell culture model in numerous studies including apoptosis [58], [59], [60], [61], cell-cycle [15], electrophysiology [62], [63], [64], oxidative stress [65], [66], signal transduction [67], [68], [69], transcriptional regulation [65], [66], [70] and cellular transplantation [71]. However, it should be noted, that the results obtained are in adult cardiomyocytes and may differ for pre-natal or post natal myocytes. This is because, we used a genetic approach of Dicer depletion in the adult mice under the control of Myh6 promoter and Myh6 expression is higher in atria than in myocytes just before or after birth [3], [32].

Taken together, these data point towards the hypothesis that strategies targeting the management of elevated miRNA-15b in *Dicer*-deficient hearts may help avert mitochondrial dysfunction and retain cardiac function.

### Conclusions

Elevated miR-15b as an early response to Dicer depletion silenced Pim-1 kinase resulting in mitochondrial dysfunction. Rescue experiments demonstrated that suppression of miRNA-15b induction in *Dicer*-deleted adult hearts result in marked amelioration of cardiac dysfunction. The significance of this observation is heightened by the reports that both Dicer deficiency [4] and elevated miRNA-15b [5], [6] is associated with many forms of cardiomyopathy and heart failure in humans. This study points towards miR-15b being one of the key miRNAs that represents a major hub regulating heart function. A recent study demonstrates that specific targeting of miR-15b alone has yielded positive results in protecting the heart against ischemic injury [72]. Since Dicer deficiency is common in other forms of heart failure, strategies to antagonize elevated miRNA-15b in the ailing heart may therefore present a broader scope and represent an efficient therapeutic approach which is worthy of consideration for clinical development.

## Supporting Information

Figure S1 **Increase in left ventricular internal diameter in Dicer^−/−^ hearts.** Quantification of LV internal diameter by ECHO.
(TIF)

Figure S2 **Initial signs of onset of fibrosis in Dicer^−/−^ hearts.** A) and C) represent Mason’s Trichrome staining of the entire heart wile B) and D) shows a corresponding zoomed region (black box). Arrows indicate collagen deposition (blue).
(TIF)

Figure S3 **Sequences.** A) MicroRNA mimic negative control, B) microRNA-15b mimic, C) siDicer, D) siControl.
(TIF)

Figure S4 **LNA™ microRNA inhibitor negative control cannot silence miR-15b in Dicer depleted animals.**
(TIF)

Movie S1 **Movie showing impaired cardiac function in Dicer−/− mice and rescue of function upon antimiRNA-15b treatment. Long axis MRI.**
(MOV)

Movie S2 **Movie showing impaired cardiac function in Dicer−/− mice and rescue of function upon antimiRNA-15b treatment. Short axis MRI.**
(MOV)

## References

[1] Zhao Y, Ransom JF, Li A, Vedantham V, von Drehle M, et al (2007) Dysregulation of cardiogenesis, cardiac conduction, and cell cycle in mice lacking miRNA-1–2. Cell 129: 303–317. 17397913 10.1016/j.cell.2007.03.030

[2] Saxena A, Tabin CJ (2010) miRNA-processing enzyme Dicer is necessary for cardiac outflow tract alignment and chamber septation. Proc Natl Acad Sci U S A 107: 87–91. 20018673 10.1073/pnas.0912870107 PMC2806718

[3] da Costa Martins PA, Bourajjaj M, Gladka M, Kortland M, van Oort RJ, et al (2008) Conditional dicer gene deletion in the postnatal myocardium provokes spontaneous cardiac remodeling. Circulation 118: 1567–1576. 18809798 10.1161/CIRCULATIONAHA.108.769984

[4] Chen JF, Murchison EP, Tang R, Callis TE, Tatsuguchi M, et al (2008) Targeted deletion of Dicer in the heart leads to dilated cardiomyopathy and heart failure. Proc Natl Acad Sci U S A 105: 2111–2116. 18256189 10.1073/pnas.0710228105 PMC2542870

[5] Small EM, Frost RJ, Olson EN (2010) MicroRNAs add a new dimension to cardiovascular disease. Circulation 121: 1022–1032. 20194875 10.1161/CIRCULATIONAHA.109.889048 PMC2847432

[6] Divakaran V, Mann DL (2008) The emerging role of microRNAs in cardiac remodeling and heart failure. Circ Res 103: 1072–1083. 18988904 10.1161/CIRCRESAHA.108.183087 PMC3982911

[7] Gnyawali SC, Roy S, McCoy M, Biswas S, Sen CK (2009) Remodeling of the ischemia-reperfused murine heart: 11.7-T cardiac magnetic resonance imaging of contrast-enhanced infarct patches and transmurality. Antioxid Redox Signal 11: 1829–1839. 19450139 10.1089/ars.2009.2635 PMC2872241

[8] Gnyawali SC, Roy S, Driggs J, Khanna S, Ryan T, et al.. (2010) High-frequency high-resolution echocardiography: first evidence on non-invasive repeated measure of myocardial strain, contractility, and mitral regurgitation in the ischemia-reperfused murine heart. J Vis Exp. 10.3791/1781 PMC3145333 20644513

[9] Zhu X, Zuo L, Cardounel AJ, Zweier JL, He G (2007) Characterization of in vivo tissue redox status, oxygenation, and formation of reactive oxygen species in postischemic myocardium. Antioxid Redox Signal 9: 447–455. 17280486 10.1089/ars.2006.1389

[10] Khanna S, Roy S, Ryu H, Bahadduri P, Swaan PW, et al (2003) Molecular basis of vitamin E action: tocotrienol modulates 12-lipoxygenase, a key mediator of glutamate-induced neurodegeneration. J Biol Chem 278: 43508–43515. 12917400 10.1074/jbc.M307075200 PMC1910692

[11] Khanna S, Roy S, Slivka A, Craft TK, Chaki S, et al (2005) Neuroprotective properties of the natural vitamin E alpha-tocotrienol. Stroke 36: 2258–2264. 16166580 10.1161/01.STR.0000181082.70763.22 PMC1829173

[12] Sen CK, Khanna S, Babior BM, Hunt TK, Ellison EC, et al (2002) Oxidant-induced vascular endothelial growth factor expression in human keratinocytes and cutaneous wound healing. J Biol Chem 277: 33284–33290. 12068011 10.1074/jbc.M203391200

[13] Sen CK, Khanna S, Roy S, Packer L (2000) Molecular basis of vitamin E action. Tocotrienol potently inhibits glutamate-induced pp60(c-Src) kinase activation and death of HT4 neuronal cells. J Biol Chem 275: 13049–13055. 10777609 10.1074/jbc.275.17.13049

[14] Crouser ED, Julian MW, Blaho DV, Pfeiffer DR (2002) Endotoxin-induced mitochondrial damage correlates with impaired respiratory activity. Crit Care Med 30: 276–284. 11889292 10.1097/00003246-200202000-00002

[15] Claycomb WC, Lanson NA Jr, Stallworth BS, Egeland DB, Delcarpio JB, et al (1998) HL-1 cells: a cardiac muscle cell line that contracts and retains phenotypic characteristics of the adult cardiomyocyte. Proc Natl Acad Sci U S A 95: 2979–2984. 9501201 10.1073/pnas.95.6.2979 PMC19680

[16] Shilo S, Roy S, Khanna S, Sen CK (2008) Evidence for the involvement of miRNA in redox regulated angiogenic response of human microvascular endothelial cells. Arterioscler Thromb Vasc Biol 28: 471–477. 18258815 10.1161/ATVBAHA.107.160655

[17] Hussain SR, Lucas DM, Johnson AJ, Lin TS, Bakaletz AP, et al (2008) Flavopiridol causes early mitochondrial damage in chronic lymphocytic leukemia cells with impaired oxygen consumption and mobilization of intracellular calcium. Blood 111: 3190–3199. 18192508 10.1182/blood-2007-10-115733 PMC2265456

[18] Reid AB, Kurten RC, McCullough SS, Brock RW, Hinson JA (2005) Mechanisms of acetaminophen-induced hepatotoxicity: role of oxidative stress and mitochondrial permeability transition in freshly isolated mouse hepatocytes. J Pharmacol Exp Ther 312: 509–516. 15466245 10.1124/jpet.104.075945

[19] Khanna S, Roy S, Parinandi NL, Maurer M, Sen CK (2006) Characterization of the potent neuroprotective properties of the natural vitamin E alpha-tocotrienol. J Neurochem 98: 1474–1486. 16923160 10.1111/j.1471-4159.2006.04000.x PMC1847628

[20] Du H, Guo L, Fang F, Chen D, Sosunov AA, et al (2008) Cyclophilin D deficiency attenuates mitochondrial and neuronal perturbation and ameliorates learning and memory in Alzheimer's disease. Nat Med 14: 1097–1105. 18806802 10.1038/nm.1868 PMC2789841

[21] Kuhn DE, Roy S, Radtke J, Gupta S, Sen CK (2006) Laser microdissection and pressure-catapulting technique to study gene expression in the reoxygenated myocardium. Am J Physiol Heart Circ Physiol 290: H2625–2632. 16443670 10.1152/ajpheart.01346.2005

[22] Kuhn DE, Roy S, Radtke J, Khanna S, Sen CK (2007) Laser microdissection and capture of pure cardiomyocytes and fibroblasts from infarcted heart regions: perceived hyperoxia induces p21 in peri-infarct myocytes. Am J Physiol Heart Circ Physiol 292: H1245–1253. 17158647 10.1152/ajpheart.01069.2006

[23] Bernstein E, Kim SY, Carmell MA, Murchison EP, Alcorn H, et al (2003) Dicer is essential for mouse development. Nat Genet 35: 215–217. 14528307 10.1038/ng1253

[24] Yang WJ, Yang DD, Na S, Sandusky GE, Zhang Q, et al (2005) Dicer is required for embryonic angiogenesis during mouse development. J Biol Chem 280: 9330–9335. 15613470 10.1074/jbc.M413394200

[25] Sohal DS, Nghiem M, Crackower MA, Witt SA, Kimball TR, et al (2001) Temporally regulated and tissue-specific gene manipulations in the adult and embryonic heart using a tamoxifen-inducible Cre protein. Circ Res 89: 20–25. 11440973 10.1161/hh1301.092687

[26] Dhalla AK, Hill MF, Singal PK (1996) Role of oxidative stress in transition of hypertrophy to heart failure. J Am Coll Cardiol 28: 506–514. 8800132 10.1016/0735-1097(96)00140-4

[27] Seddon M, Looi YH, Shah AM (2007) Oxidative stress and redox signalling in cardiac hypertrophy and heart failure. Heart 93: 903–907. 16670100 10.1136/hrt.2005.068270 PMC1994416

[28] Tsutsui H, Ide T, Kinugawa S (2006) Mitochondrial oxidative stress, DNA damage, and heart failure. Antioxid Redox Signal 8: 1737–1744. 16987026 10.1089/ars.2006.8.1737

[29] Narula N, Zaragoza MV, Sengupta PP, Li P, Haider N, et al (2011) Adenine nucleotide translocase 1 deficiency results in dilated cardiomyopathy with defects in myocardial mechanics, histopathological alterations, and activation of apoptosis. JACC Cardiovasc Imaging 4: 1–10. 21232697 10.1016/j.jcmg.2010.06.018 PMC4023693

[30] Miyamoto S, Murphy AN, Brown JH (2008) Akt mediates mitochondrial protection in cardiomyocytes through phosphorylation of mitochondrial hexokinase-II. Cell Death Differ 15: 521–529. 18064042 10.1038/sj.cdd.4402285

[31] White SM, Constantin PE, Claycomb WC (2004) Cardiac physiology at the cellular level: use of cultured HL-1 cardiomyocytes for studies of cardiac muscle cell structure and function. Am J Physiol Heart Circ Physiol 286: H823–829. 14766671 10.1152/ajpheart.00986.2003

[32] Thum T (2008) Cardiac dissonance without conductors: how dicer depletion provokes chaos in the heart. Circulation 118: 1524–1527. 18838573 10.1161/CIRCULATIONAHA.108.807230

[33] van Rooij E, Sutherland LB, Qi X, Richardson JA, Hill J, et al (2007) Control of stress-dependent cardiac growth and gene expression by a microRNA. Science 316: 575–579. 17379774 10.1126/science.1139089

[34] Rainer J, Ploner C, Jesacher S, Ploner A, Eduardoff M, et al (2009) Glucocorticoid-regulated microRNAs and mirtrons in acute lymphoblastic leukemia. Leukemia 23: 746–752. 19148136 10.1038/leu.2008.370

[35] Gantier MP, McCoy CE, Rusinova I, Saulep D, Wang D, et al (2011) Analysis of microRNA turnover in mammalian cells following Dicer1 ablation. Nucleic Acids Res 39: 5692–5703. 21447562 10.1093/nar/gkr148 PMC3141258

[36] Kai ZS, Pasquinelli AE (2010) MicroRNA assassins: factors that regulate the disappearance of miRNAs. Nat Struct Mol Biol 17: 5–10. 20051982 10.1038/nsmb.1762 PMC6417416

[37] Rissland OS, Hong SJ, Bartel DP (2011) MicroRNA destabilization enables dynamic regulation of the miR-16 family in response to cell-cycle changes. Mol Cell 43: 993–1004. 21925387 10.1016/j.molcel.2011.08.021 PMC3202612

[38] Bail S, Swerdel M, Liu H, Jiao X, Goff LA, et al (2010) Differential regulation of microRNA stability. RNA 16: 1032–1039. 20348442 10.1261/rna.1851510 PMC2856875

[39] Kosaka N, Iguchi H, Yoshioka Y, Takeshita F, Matsuki Y, et al (2010) Secretory mechanisms and intercellular transfer of microRNAs in living cells. J Biol Chem 285: 17442–17452. 20353945 10.1074/jbc.M110.107821 PMC2878508

[40] Montecalvo A, Larregina AT, Shufesky WJ, Beer Stolz D, Sullivan ML, et al.. (2011) Mechanism of transfer of functional microRNAs between mouse dendritic cells via exosomes. Blood. 10.1182/blood-2011-02-338004 PMC3265200 22031862

[41] Katakowski M, Buller B, Wang X, Rogers T, Chopp M (2010) Functional microRNA is transferred between glioma cells. Cancer Res 70: 8259–8263. 20841486 10.1158/0008-5472.CAN-10-0604 PMC2970756

[42] Roy S, Khanna S, Rink T, Radtke J, Williams WT, et al (2007) P21waf1/cip1/sdi1 as a central regulator of inducible smooth muscle actin expression and differentiation of cardiac fibroblasts to myofibroblasts. Mol Biol Cell 18: 4837–4846. 17881730 10.1091/mbc.E07-03-0270 PMC2096602

[43] Roy S, Khanna S, Hussain SR, Biswas S, Azad A, et al (2009) MicroRNA expression in response to murine myocardial infarction: miR-21 regulates fibroblast metalloprotease-2 via phosphatase and tensin homologue. Cardiovasc Res 82: 21–29. 19147652 10.1093/cvr/cvp015 PMC2652741

[44] Roy S, Khanna S, Azad A, Schnitt R, He G, et al (2010) Fra-2 mediates oxygen-sensitive induction of transforming growth factor beta in cardiac fibroblasts. Cardiovasc Res 87: 647–655. 20427335 10.1093/cvr/cvq123 PMC2920807

[45] Muraski JA, Rota M, Misao Y, Fransioli J, Cottage C, et al (2007) Pim-1 regulates cardiomyocyte survival downstream of Akt. Nat Med 13: 1467–1475. 18037896 10.1038/nm1671

[46] Fischer KM, Cottage CT, Wu W, Din S, Gude NA, et al (2009) Enhancement of myocardial regeneration through genetic engineering of cardiac progenitor cells expressing Pim-1 kinase. Circulation 120: 2077–2087. 19901187 10.1161/CIRCULATIONAHA.109.884403 PMC2787902

[47] Cottage CT, Bailey B, Fischer KM, Avitable D, Collins B, et al (2010) Cardiac progenitor cell cycling stimulated by pim-1 kinase. Circ Res 106: 891–901. 20075333 10.1161/CIRCRESAHA.109.208629 PMC3116713

[48] Borillo GA, Mason M, Quijada P, Volkers M, Cottage C, et al.. (2010) Pim-1 Kinase Protects Mitochondrial Integrity in Cardiomyocytes. Circ Res. 10.1161/CIRCRESAHA.109.212035 PMC2864233 20203306

[49] Muraski JA, Fischer KM, Wu W, Cottage CT, Quijada P, et al (2008) Pim-1 kinase antagonizes aspects of myocardial hypertrophy and compensation to pathological pressure overload. Proc Natl Acad Sci U S A 105: 13889–13894. 18784362 10.1073/pnas.0709135105 PMC2544549

[50] Sussman MA (2009) Mitochondrial integrity: preservation through Akt/Pim-1 kinase signaling in the cardiomyocyte. Expert Rev Cardiovasc Ther 7: 929–938. 19673671 10.1586/erc.09.48 PMC4066730

[51] Lilly M, Sandholm J, Cooper JJ, Koskinen PJ, Kraft A (1999) The PIM-1 serine kinase prolongs survival and inhibits apoptosis-related mitochondrial dysfunction in part through a bcl-2-dependent pathway. Oncogene 18: 4022–4031. 10435626 10.1038/sj.onc.1202741

[52] Li S, Xi Y, Zhang H, Wang Y, Wang X, et al (2010) A pivotal role for Pim-1 kinase in esophageal squamous cell carcinoma involving cell apoptosis induced by reducing Akt phosphorylation. Oncol Rep 24: 997–1004. 20811681

[53] Nishi H, Ono K, Iwanaga Y, Horie T, Nagao K, et al (2010) MicroRNA-15b modulates cellular ATP levels and degenerates mitochondria via Arl2 in neonatal rat cardiac myocytes. J Biol Chem 285: 4920–4930. 20007690 10.1074/jbc.M109.082610 PMC2836096

[54] Paradkar PN, Zumbrennen KB, Paw BH, Ward DM, Kaplan J (2009) Regulation of mitochondrial iron import through differential turnover of mitoferrin 1 and mitoferrin 2. Mol Cell Biol 29: 1007–1016. 19075006 10.1128/MCB.01685-08 PMC2643804

[55] Scorrano L, Ashiya M, Buttle K, Weiler S, Oakes SA, et al (2002) A distinct pathway remodels mitochondrial cristae and mobilizes cytochrome c during apoptosis. Dev Cell 2: 55–67. 11782314 10.1016/s1534-5807(01)00116-2

[56] Singh MK, Lu MM, Massera D, Epstein JA (2011) MicroRNA-processing enzyme Dicer is required in epicardium for coronary vasculature development. J Biol Chem 286: 41036–41045. 21969379 10.1074/jbc.M111.268573 PMC3220479

[57] Humphreys DT, Hynes CJ, Patel HR, Wei GH, Cannon L, et al (2012) Complexity of murine cardiomyocyte miRNA biogenesis, sequence variant expression and function. PLoS One 7: e30933. 22319597 10.1371/journal.pone.0030933 PMC3272019

[58] Carlson DL, Lightfoot E Jr, Bryant DD, Haudek SB, Maass D, et al (2002) Burn plasma mediates cardiac myocyte apoptosis via endotoxin. Am J Physiol Heart Circ Physiol 282: H1907–1914. 11959658 10.1152/ajpheart.00393.2001

[59] Fryer RM, Hsu AK, Nagase H, Gross GJ (2000) Opioid-induced cardioprotection against myocardial infarction and arrhythmias: mitochondrial versus sarcolemmal ATP-sensitive potassium channels. J Pharmacol Exp Ther 294: 451–457. 10900218

[60] George CH, Higgs GV, Lai FA (2003) Ryanodine receptor mutations associated with stress-induced ventricular tachycardia mediate increased calcium release in stimulated cardiomyocytes. Circ Res 93: 531–540. 12919952 10.1161/01.RES.0000091335.07574.86

[61] Gonzalez-Juanatey JR, Iglesias MJ, Alcaide C, Pineiro R, Lago F (2003) Doxazosin induces apoptosis in cardiomyocytes cultured in vitro by a mechanism that is independent of alpha1-adrenergic blockade. Circulation 107: 127–131. 12515754 10.1161/01.cir.0000043803.20822.d1

[62] Akhavan A, Atanasiu R, Shrier A (2003) Identification of a COOH-terminal segment involved in maturation and stability of human ether-a-go-go-related gene potassium channels. J Biol Chem 278: 40105–40112. 12885765 10.1074/jbc.M307837200

[63] Lanson NA Jr, Glembotski CC, Steinhelper ME, Field LJ, Claycomb WC (1992) Gene expression and atrial natriuretic factor processing and secretion in cultured AT-1 cardiac myocytes. Circulation 85: 1835–1841. 1315221 10.1161/01.cir.85.5.1835

[64] Sartiani L, Bochet P, Cerbai E, Mugelli A, Fischmeister R (2002) Functional expression of the hyperpolarization-activated, non-selective cation current I(f) in immortalized HL-1 cardiomyocytes. J Physiol 545: 81–92. 12433951 10.1113/jphysiol.2002.021535 PMC2290645

[65] Kitta K, Clement SA, Remeika J, Blumberg JB, Suzuki YJ (2001) Endothelin-1 induces phosphorylation of GATA-4 transcription factor in the HL-1 atrial-muscle cell line. Biochem J 359: 375–380. 11583584 10.1042/0264-6021:3590375 PMC1222156

[66] Kitta K, Day RM, Ikeda T, Suzuki YJ (2001) Hepatocyte growth factor protects cardiac myocytes against oxidative stress-induced apoptosis. Free Radic Biol Med 31: 902–910. 11585709 10.1016/s0891-5849(01)00663-3

[67] Nguyen SV, Claycomb WC (1999) Hypoxia regulates the expression of the adrenomedullin and HIF-1 genes in cultured HL-1 cardiomyocytes. Biochem Biophys Res Commun 265: 382–386. 10558876 10.1006/bbrc.1999.1674

[68] Sanders DB, Larson DF, Hunter K, Gorman M, Yang B (2001) Comparison of tumor necrosis factor-alpha effect on the expression of iNOS in macrophage and cardiac myocytes. Perfusion 16: 67–74. 10.1177/026765910101600110 11192310

[69] Seymour EM, Wu SY, Kovach MA, Romano MA, Traynor JR, et al (2003) HL-1 myocytes exhibit PKC and K(ATP) channel-dependent delta opioid preconditioning. J Surg Res 114: 187–194. 14559445 10.1016/s0022-4804(03)00248-8

[70] Dai B, Saada N, Echetebu C, Dettbarn C, Palade P (2002) A new promoter for alpha1C subunit of human L-type cardiac calcium channel Ca(V)1.2. Biochem Biophys Res Commun 296: 429–433. 12163037 10.1016/s0006-291x(02)00894-x

[71] Watanabe E, Smith DM Jr, Delcarpio JB, Sun J, Smart FW, et al (1998) Cardiomyocyte transplantation in a porcine myocardial infarction model. Cell Transplant 7: 239–246. 9647433 10.1177/096368979800700302

[72] Hullinger TG, Montgomery RL, Seto AG, Dickinson BA, Semus HM, et al.. (2011) Inhibition of miR-15 Protects Against Cardiac Ischemic Injury. Circ Res. 10.1161/CIRCRESAHA.111.244442 PMC3354618 22052914

