# Supplementary figures and images for: Suppression of Induced microRNA-15b Prevents Rapid Loss of Cardiac Function in a Dicer Depleted Model of Cardiac Dysfunction
Source: PLoS One. 2013 Jun 19;8(6):e66789. doi: 10.1371/journal.pone.0066789 (PMC3686742; doi:10.1371/journal.pone.0066789)

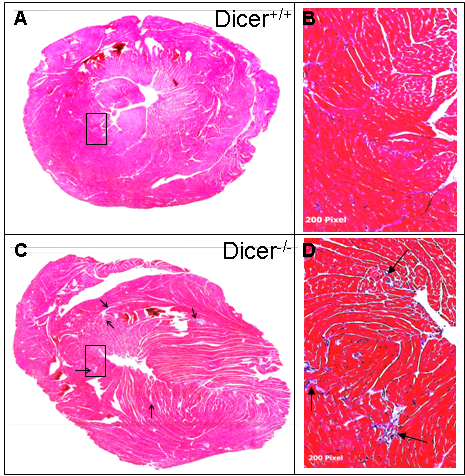

Supplement: Figure S2 — Initial signs of onset of fibrosis in Dicer−/− hearts. A) and C) represent Mason’s Trichrome staining of the entire heart wile B) and D) shows a corresponding zoomed region (black box). Arrows indicate collagen deposition (blue). (TIF) [file pone.0066789.s002.tif]
